# A New Fluorescence-Based Reporter Gene Vector as a Tool for Analyzing and Fishing Cells with Activated Wnt Signaling Pathway

**DOI:** 10.1155/2013/603129

**Published:** 2013-08-28

**Authors:** Johanna Apfel, Patricia Reischmann, Oliver Müller

**Affiliations:** University of Applied Science Kaiserslautern, 66482 Zweibrücken, Germany

## Abstract

The dysregulated Wnt pathway is a major cause for the activation of cell proliferation and reduced differentiation in tumor cells. Therefore the Wnt signaling pathway is the on-top target in searching for new anticancer drugs or therapeutic strategies. Although the key players of the pathway are known, no specific anti-Wnt drug entered a clinical trial by now. Several screening approaches for potential compounds have been performed with a reporter gene assay using multiple T-cell factor/lymphoid enhancer factor (TCF/LEF) binding motifs as promoters which control luciferase or **β**-galactosidase as reporter genes. In our work, we designed a reporter gene construct which anchors the enhanced green fluorescent protein (eGFP) to the plasma membrane. HEK 293T cells, which were stably transfected with this construct, express eGFP on the outer membrane after activation with either LiCl or WNT3A protein. Thus, cells with activated Wnt pathway could be identified and fished out of a heterogeneous cell pool by the use of magnetic-labeled anti-GFP antibodies. In summary, we present a new tool to easily detect, quantify, and sort cells with activated Wnt signaling pathway in a simple, fast, and cost-effective way.

## 1. Introduction

The dysregulated Wnt signaling pathway is linked with cancer diseases and is also one of the most mutated signaling pathways in colorectal cancer carcinomas  [1–3]. In a normal cell, the key protein *β*-catenin is permanently expressed, bound to the APC protein in the destruction complex, and marked for degradation by GSK3*β* and *β*-TrCP. As a consequence the level of free *β*-catenin is low  [4]. With activation of the canonical Wnt signaling pathway by extracellular ligands like the Wnt proteins or by mutation of one of the destruction complex proteins and due to the following misregulation *β*-catenin is translocated into the nucleus and activates expression of target genes, resulting, for example, in increased cell proliferation, reduced apoptosis, or decreased cell differentiation  [5, 6]. This is supposed to be one of the major steps in carcinogenesis from a normal cell to a tumor cell  [7].

 Specific drugs which interrupt this misregulation might have high potential in targeted cancer therapy with low side effects especially in the aggressive chemotherapies  [8, 9]. The searching for specific Wnt pathway inhibitors as possible cancer drugs forged ahead during the last years although none of them finished clinical trials studies until now  [10]. Most assays which are used to screen for Wnt modulating drugs are based on Korinek's TOPFLASH reporter gene system [11], where a luciferase enzyme activity is controlled by multiple TCF binding sites (T-cell factor), for example, three [11], six [12], seven [13], or more. Another approach uses lentiviral expression or transformation protocols to gain faster transfection results  [13–15]. These reporter gene systems open the possibility either to quantify Wnt signaling activation or to image Wnt pathway activity, respectively  [16].

Here we present a new and easy method to image, quantify, and capture Wnt-positive cells out of a pool of cells. The method is based on the antibody fishing of cells presenting a TOP-regulated reporter protein eGFP on the cell surface.

## 2. Materials and Methods

### 2.1. Cloning of pDisplay-SuperTop4-1-5 (pDis-STE4-1-5) and pDisplay-eGFP

**Figure 1:**
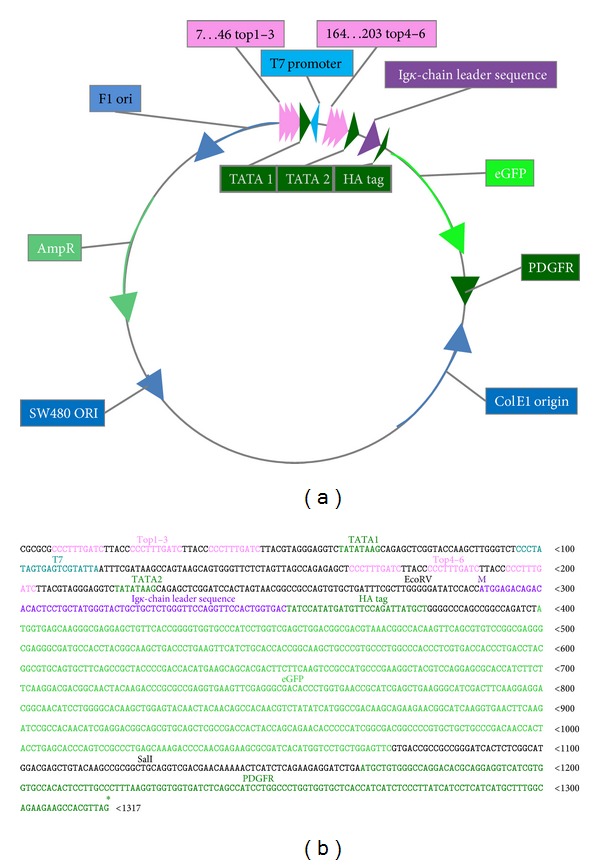
Vector map of the reporter construct pDisplay-SuperTop-eGFP4-1-5 (a) and the sequence of the cloned section (b) starting from TOP promoters to end of PDGFR sequence (M specifies start codon methionine; *describes stop codon of the expressed protein).

As initial vector the pDisplay vector construct (Invitrogen Life Technologies, Carlsbad, USA) was used. Recombinant proteins expressed from this vector are fused to the Ig*κ*-chain leader sequence, which directs the protein to the secretory pathway, and to the platelet derived growth factor receptor (PDGFR) transmembrane domain, which anchors the protein to the plasma membrane, displaying it on the extracellular side. The construct ΔTop-pDisplay was kindly provided by Dr. Sonja Eberth (University Göttingen, Germany). In this construct the pCMV promotor was replaced by three TOP motifs (5′-CCCTTTGATC-3′). Using restriction digest (SacI) and PCR (3 × Top_SacI_for: 5′-GAGCTCCCTTTGATCTTACCC-3′, pDSeq_rev: 5′-GAGGAGTGTGTCTGTCTCCAT-3′) the number of these motifs was doubled to gain 6 TCF binding motifs. The enhanced green fluorescence protein (eGFP) sequence was inserted into the multiple cloning site subsequence and in frame with the lg*κ*-chain leader sequence and PDGFR domain by using restriction digest with PspOMI and SalI (Figure 1).

The positive control vector pDisplay-eGFP was generated by inserting the eGFP gene sequence into the pDisplay vector using restriction digest (EcoRV, SalI). In cells transfected with this vector, eGFP is expressed constitutively under the pCMV promoter.

Vectors were transformed into *E. coli* DH5*α* following standard protocols. All restriction enzymes and bacteria for transformation were obtained from New England Biolabs (Frankfurt, Germany).

### 2.2. Cell Culture

For proof-of-principle experiments, the human embryonal kidney cells HEK293T were used because of their good transfection results. They were cultured in Dulbecco's Modified Eagle Medium (DMEM) supplemented with 10% fetal calf serum (PAN Biotech, Aidenbach, Germany), 1% Penicillin/Streptomycin, and 1% nonessential amino acids (Sigma Aldrich, Munich, Germany) with 5% CO_2_ at 37°C. Media were changed every two days; subcultivation was done with a visual confluence of 70%.

### 2.3. Transfection

All transfections were carried out with the lipofection reagent Roti-Fect Plus (Carl Roth, Karlsruhe, Germany) due to the manufacturers' instructions.

For stable transfection, cells were seeded onto six-well plates 48 hours prior to transfection. For transfection, either 1 *μ*g pDis-STE4-1-5 or pDisplay-eGFP plasmid was used. The transfection media were changed with completed media 16 hours after transfection. 48 hours after transfection cells were subcultivated into a new plate and media were supplemented with 1 mg/mL Geneticin (G418, Carl Roth, Karlsruhe, Germany) until nontransfected control cells died (approximately 3 weeks). 

### 2.4. Microscopic Analysis

HEKT-STE4-1-5 cells were seeded into 24-well plates and treated with different concentrations of LiCl (0,01 mM, 0,1 mM, 1 mM, and 10 mM) or WNT3A (20 ng/mL, 50 ng/mL, and 100 ng/mL). Wnt-specific response was monitored via fluorescence microscopy (Observer.Z1 from Zeiss, Jena, Germany) and the associated software AxioVision4.7 24 hours and 48 hours after addition of the activating agent.

### 2.5. Western Blot Analysis

For preparation of Western Blot samples, up to 5 × 10^6^ cells per sample were trypsinized, centrifuged, and resuspended in 0,5 mL of 0,1 × PBS supplemented with protease inhibitor. Samples were centrifuged for 4 hours at 13,000 rpm and 4°C. Supernatant (cytoplasm) and pellet (membrane fraction) were used for Western Blot analysis. After adjustment of protein concentrations, lysates were boiled in SDS sample loading buffer for 5 minutes and separated by SDS-polyacrylamide gel electrophoresis (NuPAGE, 4–12%, Invitrogen). Gel was blotted onto a nitrocellulose membrane via iBlot transfer system (Invitrogen) and stained with the anti-GFP, anti-beta-catenin (6B3) first antibody (diluted 1 : 1000 in TBS-Tween with 3% milk, CellSignaling), or anti-phospho-beta-catenin antibody (S33/37/T41) (diluted 1 : 1000 in TBS-Tween). Antibody binding was detected with a horseradish-peroxidase- (HRP-) coupled secondary antibody (dilution 1 : 2000) followed by chemoluminescence detection (RotiLumin, Carl Roth, Karlsruhe, Germany).

### 2.6. Quantitative Determination Assay

Forty-eight hours before the assay HEKT-STE4-1-5 1C cells were seeded into fluorescence-compatible 96-well plates at a density of 1,5 × 10^5^ cells per well. Assays were carried out in phenol red-free DMEM supplemented as described above. Cells were treated with 0,01 mM, 0,1 mM, 1 mM, or 10 mM LiCl or 20 ng/mL, 50 ng/mL, or 100 ng/mL WNT3A protein. The fluorescence intensity was measured in an ELISA reader (Tecan GENios, Männedorf, Switzerland) immediately, 24 h and 48 h after treatment using standard GFP filter sets. Fluorescence measurement was coupled with cell viability assay WST-1 (Roche, Mannheim, Germany) according to manufacturer's manual. Data were referred to the cell number and normalized to the untreated control. Data are reported as means ± SD of at least three independent experiments with each in triplicate.

### 2.7. Cell Sorting with MACS System

Fishing of cells with activated Wnt signaling pathway was carried out with the MACS (magnetic activated cell sorting) system using LD columns and *μ*MACS anti-GFP beads which were kindly provided by Miltenyi Biotech, Bergisch Gladbach, Germany.

HEKT-STE4-1-5 cells were partly treated with 5 mM LiCl. Twenty-four hours later cells were counted and 5 × 10^6^ cells in single cell suspension were applied for the separation. After centrifugation (1000 rpm for 5 min) supernatant was aspirated completely and cells were suspended in 80 *μ*L of cold MACS buffer (PBS supplemented with 1% BSA and 2 mM EDTA) and suspended with 20 *μ*L anti-GFP beads. After incubation (4°C, 25 min) suspensions were washed by adding 1 mL MACS buffer and centrifugation for 10 min at 300 ×g. Supernatant was aspirated and cells were resuspended in 2 mL MACS buffer.

For magnetic separation MACS LD columns were placed in a magnetic field and rinsed with 3 mL MACS buffer for equilibration. The cell suspension was applied onto the column and the unlabeled flowthrough was collected. Columns were washed two times with each 3 mL MACS buffer. For collecting labeled cells the columns were removed from the magnetic field and applied with 5 mL MACS buffer. The cells were flushed out by firmly pushing the plunger into the column. The number of cells in each collected fraction was determined by the use of Neubauer counting chamber. The first flowthrough and the labeled fraction were seeded out in a cell flask and cultivated as described. Cell viability and fluorescence were observed every 24 hours after separation up to 3 days.

## 3. Results 

### 3.1. Generation of Stably Transfected Cells

The cloned inserts for both pDis-STE4-1-5 and pDisplay-eGFP plasmids were sequenced (GATC, Heidelberg, Germany). Figure 1 shows the vector map (a) and the sequence cutout from origin of replication to eGFP start codon (b) of the vector pDis-STE4-1-5. 

In order to test the Wnt pathway reactivity both plasmids were stably transfected into HEK293T cells. Clones were characterized due to their Wnt responsiveness by applying LiCl to the clones and monitoring the increase in the fluorescence signal via microscopic analysis. The final stably transfected clones were named HEKT-STE4-1-5 1C and HEKT-eGFPA1. Transfection success was checked by PCR of the eGFP sequence (data not shown). The morphology of the new cell lines is the same as the originals except for the fluorescent signal localized to the membrane.

### 3.2. Determination of Wnt Response in HEKT-STE4-1-5 1C Cells

**Figure 2:**
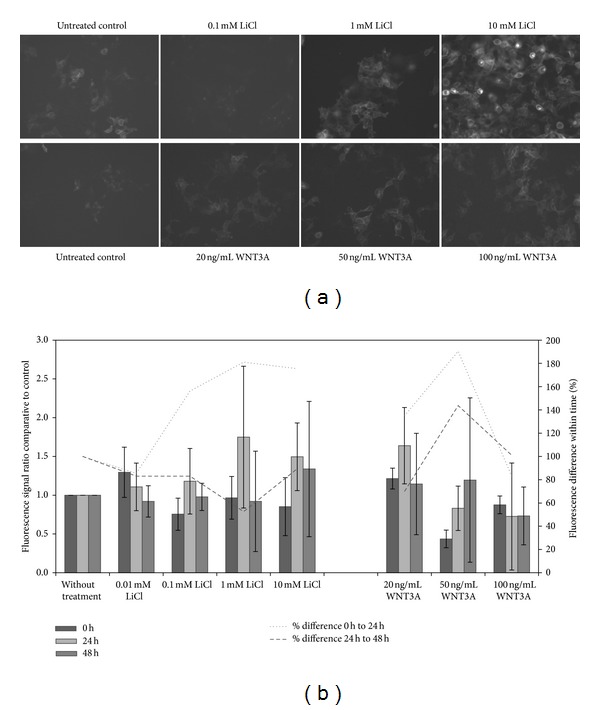
Results of the characterization of the vector construct SuperTop-eGFP4-1-5. (a) Microscopic analysis of HEKT-STE4-1-5 1C cells treated with either 0,1 mM, 1 mM, 10 mM LiCl (upper row, from left to right) or 20 ng/mL, 50 ng/mL, 100 ng/mL WNT3A (lower row, from left to right), respectively. Representative images were taken 24 hours after treatment. The fluorescence signal was highest with 10 mM LiCl due to Wnt pathway activation. Treatment with either concentration of WNT3A also showed minor increase in fluorescence as compared to LiCl activation. Magnification 200x. (b) Quantitative analysis of Wnt pathway activation in stably transfected HEKT-STE4-1-5 1C cells. Cells were treated either with LiCl or WNT3A protein and fluorescence signal was monitored right after, 24 hours and 48 hours after treatment. Bars are showing the fluorescence signal in comparison to the untreated control, which was normalized to 1 (left axis). Data were normalized to cell number per well. The lines show the percentage difference in fluorescence signal from 0 h to 24 hours and from 24 hours to 48 hours measurements (right axis). In LiCl-treated cells the highest fluorescence increase is displayed 24 hours after treatment and with a concentration of 1 mM LiCl. Highest change in fluorescence after addition of WNT3A was monitored within a concentration of 50 ng/mL WNT3A, although the signal is lower as compared to the signal obtained in cells treated with 20 ng/mL WNT3A which is comparative to the signal in 1 mM LiCl-treated cells.

For testing the Wnt-specific response HEKT-STE4-1-5 1C cells were seeded into 24-well plates and treated with several LiCl or WNT3A protein concentrations as described above. The fluorescence signal due to Wnt pathway activation was the highest by the addition of 10 mM LiCl and after 24 hours and still visible 48 hours after treatment (Figure 2(a)). A higher fluorescence signal is also monitored in cells treated with WNT3A although the signal magnification is not as high as in LiCl-treated cells. Even in untreated cells a slightly fluorescence signal is gathered. This might be explained by the constitutive background activation of the Wnt pathway. 

To quantify the Wnt signaling pathway activation HEKT-STE4-1-5 1C cells were seeded into 96-well plates and measured using a standard ELISA reader. Cells were treated according to fluorescence analysis experiments and the fluorescence was measured. The results demonstrate an increased fluorescence signal relative to the control within 24 hours of either LiCl or WNT3A treatment whereas concentrations below 1 mM LiCl show no effect on the fluorescence (Figure 2(b)). Concentrations above 50 ng/mL Wnt3a interestingly had no further effect. The highest signal amplification is monitored after 24 hours within 1 mM LiCl (175% compared to untreated control) and 10 mM LiCl (150% compared to untreated control). The highest increase in the fluorescence signal was obtained after 24 hours of treatment with 50 ng/mL WNT3A (190% difference to day before). 

HEKT-eGFPA1 cells bearing the constitutive pDisplay-eGFP plasmid which were tested in a control experiment showed no effect on fluorescence level at all (data not shown).

**Figure 3:**
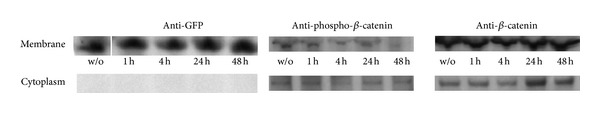
Western Blot analysis of HEKT-STE4-1-5 1C cells treated with 1 mM LiCl for 1, 4, 24, or 48 hours as indicated. eGFP protein is detected in the membrane fraction but not in the cytoplasm and slightly increased 24 and 48 hours after treatment. Highest *β*-catenin protein concentration was detected at 24 hours in the cytoplasm. Concentration of nonphosphorylated *β*-catenin in the membrane fraction remained constant, but the phosphorylated form decreased within time.

Western Blot was performed to evaluate eGFP expression on the outer cell membrane (Figure 3). Because Wnt pathway activation was the highest with LiCl, HEKT-STE4-1-5 1C cells were treated with 1 mM LiCl. eGFP protein is restricted to the membrane fraction while beta-catenin protein was detectable in both membrane and cytoplasm fraction. Furthermore, eGFP protein level was increased 24 and 48 hours after treatment, while the level of beta-catenin remained constant. 

### 3.3. Cells Bearing the New Construct Can Be Sorted Using Ant-GFP Antibodies

**Figure 4:**
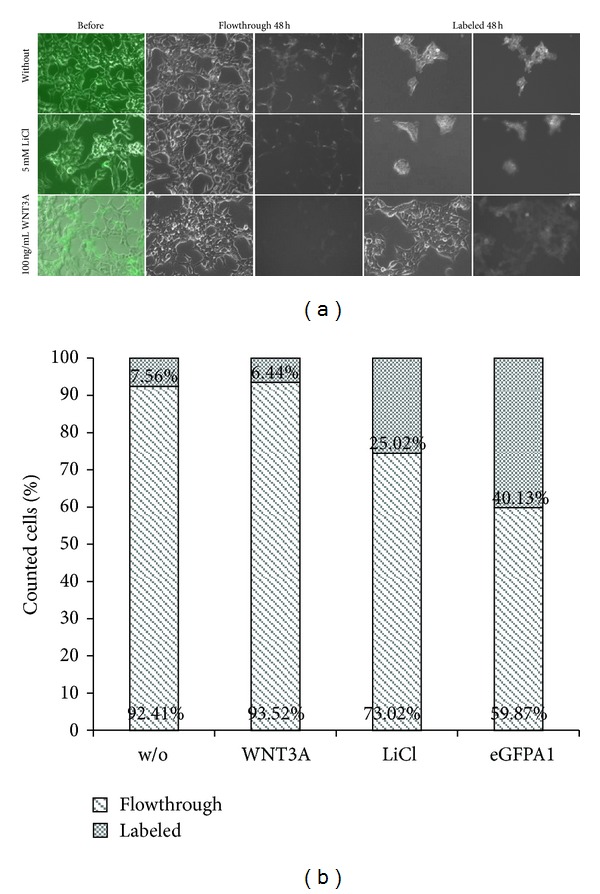
Sorting cells transfected with MACS Separation system. Cells were treated either with or without 5 mM LiCl or 100 ng/mL WNT3a in order to activate the Wnt signaling pathway. Sorting occurred 24 hours after treatment. Every fraction was collected and cultured up to 3 days. (a) Representative images of the cells before (left row; phase contrast and fluorescence are merged) and 48 hours of cultivation after sorting. Cells were sorted almost completely into activated (labeled, right row splitted in phase contrast and fluorescence) or nonactivated cells (middle row). Magnification 200x. (b) Comparison of sorted cells within different treatments. w/o: without treatment; WNT3A: 50 ng/mL; LiCl: 5 mM; eGFPA1: untreated HEKT-eGFPA1 cells as positive control. Highest sorting result was achieved with activation of HEKT-STE4-1-5 1C cells with 5 mM LiCl.

In order to “catch” cells with an activated Wnt signaling pathway, HEKT-STE4-1-5 1C cells were activated with either 5 mM LiCl or 100 ng/mL WNT3A protein and positive cells were concentrated by using the MACS separation system. The technology is based on antibodies labeled with magnetic beads and thus can be caught with a magnetic field applied to the cell sorting columns. We used the *μ*MACS anti-GFP beads kit applicable for purification of GFP pure protein and optimized the protocol for sorting cells expressing eGFP instead. The technology works for the separation of Wnt-positive and -negative cells and also for a minor Wnt activation status. This is shown by the fact that untreated control cells with spontaneously activated GFP expression were also sorted (Figure 4). Also cells were viable after separation as they could be cultured as normal. The positive sorted separation percentage was about 25% within LiCl activated cells and 6,4% among WNT3A activated cells (Figure 4(b)) which correlated to the results in the microscopic analysis and the quantitative determination assay. Nevertheless, sorting Wnt activated cells out of a pool of differentially expressed eGFP due to Wnt pathway activation worked very well. All sorted cells cultivated after sorting were viable and positive. Within the flowthrough there were only few false negatives left, what might be due to additional spontaneously Wnt pathway activation after sorting process. 

As a control, we also used nontransfected HEK293 cells or HEK293T cells transfected with pDisplay-eGFP vector. Cells without bearing the reporter construct were directly passing the magnetic column (0,28% false positive out of 5 × 10^6^ cells). Sorting constitutively expressing eGFP HEKT-eGFPA1 cells resulted in 40% positive and 60% negative cells, although all cells applied on the column were positive. This might be explained by the fact that depletion columns are not suitable for sorting 100 percent positive cells.

## 4. Discussion

Investigations on the Wnt signaling pathway and its accompanying networks became more and more important over the past decades. Up to date there are many protocols for characterizing the activation of the Wnt pathway within cells. While some might be used to gain new insights into Wnt pathway regulation  [17, 18] and its correlation to other networks, others are used in screening for new potential anti-cancer drugs  [12, 19]. In this study a new reporter gene vector is introduced which is aiming to quantify Wnt signaling state in a cell and capture it out of a cell pool at the same time. For evaluation and characterization of this reporter we used HEK 293T cells as they were reported to be high responsive to Wnt signaling activation by LiCl  [20] or WNT3A protein addition  [21]. Although their Wnt response was reported to be high by addition of WNT3A  [22] we monitored higher Wnt response in evidence of eGFP expression after treatment with LiCl. According to this effect, we used LiCl activation for all followed experiments. Highest Wnt response was monitored 24 hours after addition of LiCl. That way activated cells bearing the reporter construct are catchable in a magnetic field using magnetic-labeled GFP antibodies. 

Although the basic principle for the use of antibodies for catching cells has been described  [23], there is—according to our knowledge—neither an established protocol for the separation of cells with Wnt activation nor catching or even enrich cells using anti-GFP antibodies yet. Our results show that it is possible to detect and quantify cells with activated Wnt signaling pathway and additionally fish them out of a differentially expressing HEK293T cell pool using our reporter construct. All cells separated by using our magnetic separation strategy were viable and could be used for further cultivation. Although we investigated only transfected cell lines so far, this approach may be applied for identifying and fishing cells throughout primary cells or even for catching cells out of body fluids as part of a diagnostic procedure when using antibodies against membrane bound Wnt target gene products. During the last years circulating tumor cells (CTCs) and the necessity to capture them out of blood attracted focus. First described methods for catching those cells use tagged antibodies  [24] which are similar to our protocol. So our findings may also be an approach suitable for that application, not just for cells transfected with our plasmid but also for using Wnt target proteins which are expressed on the outer membrane (e.g., Frizzled7  [25], Nr-CAM  [26], or CD44  [27]).

In conclusion we designed a new reporter gene construct suitable for an easy, fast, and cheap characterization of cells with activated Wnt pathway and also for capturing out of a pool of cells differentially expressing the pathway. Fluorescence-based reporter gene assays are more cost effective than the current luciferase-based reporters and our assay does not need any additional, expensive lab equipment. After optimization and adaptation our protocol may be applicable as well in drug screening assays as in diagnostic procedures.
